# Inverse probability weighting to handle attrition in cohort studies: some guidance and a call for caution

**DOI:** 10.1186/s12874-022-01533-9

**Published:** 2022-02-16

**Authors:** Marie-Astrid Metten, Nathalie Costet, Luc Multigner, Jean-François Viel, Guillaume Chauvet

**Affiliations:** 1 Univ Rennes, CHU Rennes, Inserm, EHESP, Irset (Institut de Recherche en Santé, Environnement et Travail) - UMR_S 1085, Rennes, France; 2 Univ Rennes, Inserm, EHESP, Irset (Institut de Recherche en Santé, Environnement et Travail) - UMR_S 1085, Rennes, France; 3 ENSAI, CNRS, IRMAR-UMR 6625, Rennes University, F-35000 Rennes, France

**Keywords:** Cohort studies, Attrition, Missing outcome, Selection bias, Inverse probability weighting, Complete-case analysis

## Abstract

**Background:**

Attrition in cohort studies challenges causal inference. Although inverse probability weighting (IPW) has been proposed to handle attrition in association analyses, its relevance has been little studied in this context. We aimed to investigate its ability to correct for selection bias in exposure-outcome estimation by addressing an important methodological issue: the specification of the response model.

**Methods:**

A simulation study compared the IPW method with complete-case analysis (CCA) for nine response-mechanism scenarios (3 missing at random – MAR and 6 missing not at random - MNAR). Eighteen response models differing by the type of variables included were assessed.

**Results:**

The IPW method was equivalent to CCA in terms of bias and consistently less efficient in all scenarios, regardless of the response model tested. The most effective response model included only the confounding factors of the association model.

**Conclusion:**

Our study questions the ability of the IPW method to correct for selection bias in situations of attrition leading to missing outcomes. If the method is to be used, we encourage including only the confounding variables of the association of interest in the response model.

**Supplementary Information:**

The online version contains supplementary material available at 10.1186/s12874-022-01533-9.

Cohort studies are essential for investigating associations between exposure and health outcomes thanks to their prospective design. The repeated collection of information in successive follow-ups (also called survey waves) allows studying the effects of past exposures on health outcomes occurring at inclusion or thereafter. However, such studies are known to be affected by partial and total non-response, which can invalidate the causal inference that can be drawn from them. Partial non-response refers to missing data that occasionally occurs for certain variables during a survey wave when some individuals fail or refuse to answer some of the questions. Total non-response (or attrition) occurs when a subset of individuals does not participate in one specific survey wave or quit the study completely [1]. Only the latter (drop-outs) was considered in this study.

Missing data resulting from non-response can be classified according to their postulated underlying mechanism [2]. In situations of the *missing completely at random* mechanism (MCAR), the probability of missing data does not depend on either the observed or unobserved values. In situations of the *missing at random* mechanism (MAR), it depends on the observed data but not the unobserved data. Finally, in situations of the *missing not at random* mechanism (MNAR), it depends on the unobserved data.

The simplest and most widely used approach to handle total non-response in cohort studies is complete-case analysis (CCA). This method assumes a MCAR mechanism and consists of studying the exposure-outcome association in the subset of respondents only. However, total non-response is generally considered to result from MAR or MNAR mechanisms. Several methodological publications have suggested the use of the inverse probability weighting (IPW) method in situations of the MAR mechanism of attrition [3, 4]. It aims to recreate a representative sample of the initial cohort by differentially weighting the so-called “complete individuals” (i.e. those who participate in the survey wave under consideration). More precisely, when modeling the association between exposure and outcome, respondents are weighted by the inverse of their probability to participate (hereinafter referred to as the “response probability” or “probability of response”). This response probability depends on some of the respondent’s characteristics. The use of the inverse of this probability implies that a respondent with a high probability of response (e.g. an individual with a high socio-economic level [5]) is given a comparatively lower weight in the analysis. The approach can be summarized as: “the respondents carry the weight of the non-respondents”.

The probability of response is unknown and needs to be estimated from the data. The first step is therefore to build a response model (logistic regression model) to obtain weights that will be used in a second step in the association model. Because the association model is only fitted among respondents or complete individuals, the method is also called “weighted complete-case analysis” [2]. It is also referred to as “inverse probability of *participation/attrition* weighting” (IPPW/IPAW) in the literature [6–8].

Originally developed for reducing the effects of confounding in observational studies (propensity score method) [9], the IPW method was extended to correct for selection biases in situations of attrition. Although researchers have already adopted the method in association studies [10, 11], guidance on its correct use is still needed, in particular regarding the specification of the response model (i.e. variables to be introduced into the response model).

In this article, we will focus on attrition resulting in a missing outcome of interest. This situation is commonly encountered in mother-child cohorts for example, where the effects of prenatal medical conditions or exposures on the future health of the children are studied. At the time point of interest (6 year old, for example), some children do not participate in the follow-up. Depending on the attrition mechanism (MAR, MNAR), restricting the analysis to the participating children may result in a biased estimation of the association between the exposure and the outcome.

Our work aimed at evaluating through simulations the ability of the IPW method to correct for a selection bias under various missingness mechanisms and specifications of the response model. Response model specifications were compared in terms of bias, variance and mean square error of the association estimates between the exposure and the outcome.

In all scenarios tested, we assumed that the exposure variable and the other covariates were fully observed at preceding waves or at baseline.

## Which variables should be introduced into the response model?

Relatively few authors have addressed the question of which variables should be introduced in the response model from which the weighting is derived. In 2004, Hernan et al. recommended including the exposure variable and all variables that independently predict both response and outcome [3]. In 2013, Seaman and White advised not including variables that are exclusively related to the response without being related to the outcome and exposure variables. They suggested adding confounding variables (i.e. associated with both exposure and outcome) and prognostic variables (i.e. exclusively associated with the outcome) of the association studied [4]. Seaman and White’s recommendations are consistent with simulation studies performed in the propensity score literature [12, 13]. In this context, including variables that are related to the exposure but not the outcome is discouraged. Variables unrelated to the exposure but related to the outcome should instead be included. The literature on the IPEW method considers only two variables: the exposure and the outcome, whereas the IPPW method involves the response as a third variable. Thus, there is still uncertainty as to whether the inclusion of confounding and prognostic variables in the response model depends on whether or not they are associated with the response. Furthermore, if the exposure variable X is itself associated with the response, should it be included in the response model?

A quick glance at the applied literature shows that researchers usually build the response and association models independently. They often fit a logistic regression model by including the presumed predictors of response, whether or not they are related to exposure or outcome [14, 15]. Their approach is thus primarily to fit a model that perfectly predicts the response and not to optimize the response model in relation to the association model.

None of the proposed strategies in the literature has been tested through simulations and they do not appear to be applied by researchers. Therefore, we propose here to provide insight on this issue by studying the impact of the type of variables included in the response model on the bias and variance of the exposure regression coefficient in the association model.

## Simulation study

We conducted a Monte-Carlo simulation study under several MAR and MNAR scenarios. We aimed to evaluate i) the relative performance of the IPPW method relative to CCA and ii) how the specification of the response model in the IPPW method affects the bias of the exposure regression coefficient $\hat{\beta}$, its variance and mean square error, and the coverage rate of confidence intervals.

The SAS code to implement the simulation study is available in Additional file 1: e-Appendix 1.

We focused on the case of a linear regression model in which a continuous outcome is explained by continuous exposure and covariates.

### Data-generating process

We first created a sample of size *n* = 1,000, containing seven covariates *z*_1_, …, *z*_7_ generated independently according to standard normal distributions. We then generated an exposure variable according to the following model:

1 $${x}_i=1+{\alpha}_1\ {z}_{1i}+{\alpha}_2\ {z}_{2i}+{\alpha}_5\ {z}_{5i}+{\alpha}_6{z}_{6i}+{\epsilon}_i,$$

where *ϵ*_*i*_ is generated according to a standard normal distribution. In the exposure model (1), the coefficients were chosen as *α*_1_ = *α*_2_ = *α*_5_ = *α*_6_ = 0.218, so that the correlation between *x*_*i*_ and each of the covariates *z*_*i*_ was approximately 0.2. We generated an outcome variable according to the following model:

2 $${y}_i=1+\beta {x}_i+{\beta}_1\ {z}_{1i}+{\beta}_3\ {z}_{3i}+{\beta}_5\ {z}_{5i}+{\beta}_7\ {z}_{7i}+{\epsilon}_i^{\prime },$$

where *ϵ*′_*i*_ is generated according to a standard normal distribution. In the outcome model (2), the coefficients were chosen as *β* =0.5, *β*_1_ = *β*_5_ = 0.170, and *β*_3_ = *β*_7_ = 0.230, such that the correlation between *y*_*i*_ and *x*_*i*_ was approximately 0.3,and the correlation between *y*_*i*_ and any of the covariates *z*_*i*_ was approximately 0.2. Finally, we generated response probabilities according to the following logistic model:

3 $$\mathrm{logit}\left({p}_i\right)={\gamma}_0+{\gamma}_y\ {y}_i+{\gamma}_x\ {x}_i+{\gamma}_1\ {z}_{1i}+{\gamma}_2\ {z}_{2i}+{\gamma}_3\ {z}_{3i}+{\gamma}_4\ {z}_{4i},$$

**Fig. 1:**
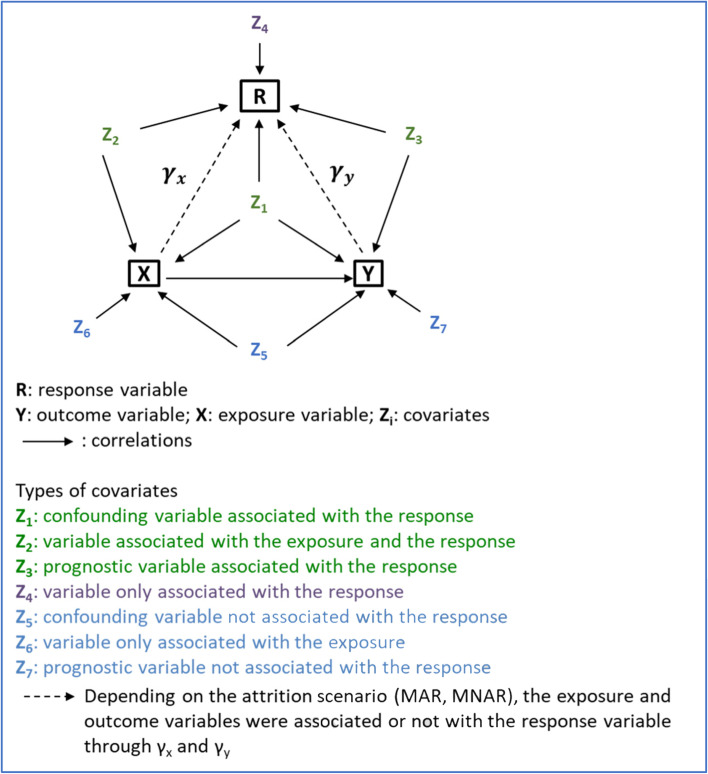
Scheme of the data-generation model for the simulation experiments. Seven covariates, differing in their association with the variables of interest (exposure and outcome variables) and the response variable, were generated. The strength of the associations (dashed arrows) between the variables of interest and the response variable (γ_x_ and γ_y_) varied according to the scenarios described in Table 1

We used the values *γ*_*y*_ = 0.0, 0.2 or 0.5 and *γ*_*x*_ = 0.0, 0.2 or 0.5. The case in which *γ*_*y*_ =0.0 corresponds to a MAR situation (i.e. the response probability does not depend on *y*_*i*_). The cases in which *γ*_*y*_ =0.2 and 0.5 correspond to MNAR situations (i.e. the response probability depends on *y*_*i*_). In the response model (3), the coefficients *γ*_1_, *γ*_2_, *γ*_3_, and *γ*_4_ were chosen to be equal to 0.1. The coefficient *γ*_0_ was chosen such that the average response rate was approximately 60% for all cases. In the sample, the individuals responded independently with the probabilities *p*_*i*_. The data-generation model is presented in Fig. 1 and the nine response mechanism scenarios are summarized in Table 1.

**Table 1:** Response mechanism scenarios (data generation)

Scenario	***γ***_***x***_	***γ***_***y***_	$${\boldsymbol{\gamma}}_{\mathbf{1}},{\boldsymbol{\gamma}}_{\mathbf{2}},{\boldsymbol{\gamma}}_{\mathbf{3}},{\boldsymbol{\gamma}}_{\begin{array}{c}\mathbf{4}\\ {}\ \end{array}}$$	Description
MAR 1	0.0	0.0	0.1	Response depending only on covariates
MAR 2	0.2	0.0	0.1	Response depending on covariates and exposure
MAR 3	0.5	0.0	0.1	Response depending on covariates and exposure
MNAR 1	0.0	0.2	0.1	Response depending on outcome and covariates
MNAR 2	0.2	0.2	0.1	Response depending on outcome, exposure, and covariates
MNAR 3	0.5	0.2	0.1	Response depending on outcome, exposure, and covariates
MNAR 4	0.0	0.5	0.1	Response depending on outcome and covariates
MNAR 5	0.2	0.5	0.1	Response depending on outcome, exposure, and covariates
MNAR 6	0.5	0.5	0.1	Response depending on outcome, exposure, and covariates

***γ***_***k***_:regression coefficients of the generated response models (logit(*p*_*i*_) = *γ*_0_ + *γ*_*y*_ *y*_*i*_ + *γ*_*x*_ *x*_*i*_ + *γ*_1_ *z*_1*i*_ + *γ*_2_ *z*_2*i*_ + *γ*_3_ *z*_3*i*_ + *γ*_4_ *z*_4*i*_)

### Simulation parameters and performance criteria

We compared the IPPW method to CCA for a parsimonious association model, i.e. including only the confounding variables Z_1_ and Z_5_, which corresponds to standard epidemiological practice: ${y}_i=1+{x}_i+{\mathrm{z}}_{1\mathrm{i}}+{z}_{5i}+{\epsilon}_i^{\prime }$.

Several response models were tested (see Table 2) to determine the impact of the type of variables included on the $\hat{\beta}$ regression coefficient of the exposure variable and its variance in the association model. Briefly, we first evaluated the “well-specified” response model, i.e. the one that included all the variables really related to the response (X, Z_1_, Z_2_, Z_3_, Z_4_), as simulated (Eq. 1). We also initially included the exposure variable X, although this variable was not associated with the response in certain tested scenarios (MAR 1, MNAR 1, MNAR 4). We then tested a model including all available variables. Then, we assessed the proposals by Hernan et al. (2004) and by Seaman and White (2013), described above [3, 4]. Finally, we evaluated parsimonious strategies: including only the confounding variable associated with the response, including only the confounding variable not associated with the response, including both, including both with the addition of a prognostic variable not associated with the response, and finally, including both confounding and prognostic variables not associated with the response. All these response models were then evaluated without the exposure variable X.

**Table 2:** Response models tested

Response model	Set of variables	Description
1	X, Z_1_, Z_2_, Z_3_, Z_4_	All variables associated with the response^a^
2	X, Z_1_, Z_2_, Z_3_, Z_4_, Z_5_, Z_6_, Z_7_	The exposure variable X and all covariates
3	X, Z_1_, Z_3_	The exposure variable X and variables associated with both response and outcome (strategy proposed by Hernan et al. [3])
4	X, Z_1_, Z_2_, Z_3_, Z_5_, Z_7_	All variables associated with the response*, except Z_4_ only associated with the response; Adding Z_5_ a confounding variable (Z_5_) and a prognostic variable (Z_7_), neither associated with the response (strategy proposed by Seaman and White [4])
5	X, Z_1_	The exposure variable X and the confounding variable associated with the response (Z_1_)
6	X, Z_5_	The exposure variable X and the confounding variable not associated with the response (Z_5_)
7	X, Z_1,_ Z_5_	The exposure variable X and both confounding variables, that associated with the response (Z_1_) the other not (Z_5_)
8	X, Z_1,_ Z_5_, Z_7_	The exposure variable X, both confounding variables (Z_1_, Z_5_) and a prognostic variable not associated with the response (Z_7_)
9	X, Z_5_, Z_7_	The exposure variable X and a confounding variable and prognostic variable, neither associated with the response (Z_5_, Z_7_)
10	Z_1_, Z_2_, Z_3_, Z_4_	Previous response models without the exposure variable X
11	Z_1_, Z_2_, Z_3_, Z_4_, Z_5_, Z_6_, Z_7_	
12	Z_1_, Z_3_	
13	Z_1_, Z_2_, Z_3_, Z_5_, Z_7_	
14	Z_1_	
15	Z_5_	
16	Z_1,_ Z_5_	
17	Z_1,_ Z_5_, Z_7_	
18	Z_5,_ Z_7_	

^a^X was not associated with the response in scenarios MAR 1, MNAR 1, or MNAR 4

The generation of the sample and variables was repeated *B* = 10,000 times. For each sample, we computed the $\hat{\beta}$ regression coefficient and its variance according to the 18 possible response models. The simulations were conducted using SAS version 9.4.

The results were assessed according to the following criteria:

- The Monte Carlo bias:
  $${B}_{MC}\left({\hat{\beta}}_x\right)=\frac{1}{\mathrm{10,000}}\sum_{b=1}^{\mathrm{10,000}}\left({\hat{\beta}}_x^b-\beta \right)$$
- The Monte Carlo variance:
  $${V}_{MC}\left({\hat{\beta}}_x\right)=\frac{1}{\mathrm{10,000}-1}\sum_{b=1}^{\mathrm{10,000}}{\left({\hat{\beta}}_x^b-\overline{\hat{\beta_x}}\right)}^2\ with\ \overline{\hat{\beta_x}}=\frac{1}{\mathrm{10,000}}\sum_{b=1}^{\mathrm{10,000}}{\hat{\beta}}_x^b.$$
- The mean square error:
  $${MSE}_{MC}\left({\hat{\beta}}_x\right)=\frac{1}{\mathrm{10,000}-1}\sum_{b=1}^{\mathrm{10,000}}{\left({\hat{\beta}}_x^b-\beta \right)}^2.$$
- The relative root mean square error:
  $${\boldsymbol{RRMSE}}_{\boldsymbol{MC}}\left({\hat{\boldsymbol{\beta}}}_{\boldsymbol{x}}\right)=\mathbf{100}\times \frac{\sqrt{{\boldsymbol{MSE}}_{\boldsymbol{MC}}\left({\hat{\boldsymbol{\beta}}}_{\boldsymbol{x}}\right)}}{\boldsymbol{\beta}}.$$

The Monte Carlo variance is the variance of the estimates over 10,000 replications. Therefore, it accounts for the entire variability of the estimators, including the fact that the weights are estimated. We have also computed the coverage rates for the normality-based confidence intervals for ${\hat{\boldsymbol{\beta}}}_{\boldsymbol{x}}$, with nominal rates of 2.5% in each tail.

## Results of the simulation study

The simulation results are reported in Tables 3, 4 and 5 for the Monte Carlo bias, variance, mean square error and relative root mean square error, and in Table 6 for the coverage rates.

**Table 3:** Simulation study results: bias, variance, mean square error and related root mean square error in the $\hat{\beta}$ regression coefficient for CCA and the IPPW method (18 response models), for three MAR response mechanism scenarios

Scenario^a^	***γ***_***x***_	***γ***_***y***_		CCA		IPPW method								
						*Response models*								
						(X), Z_1_, Z_2_, Z_3_, Z_4_	(X), Z_1_, Z_2_, Z_3_, Z_4_, Z_5_, Z_6_, Z_7_	(X), Z_1_, Z_3_	(X), Z_1_, Z_2_, Z_3_, Z_5_, Z_7_	(X), Z_1_	(X), Z_5_	(X), Z_1_, Z_5_	(X), Z_1_, Z_5_, Z_7_	(X), Z_5_, Z_7_
MAR 1	0.0	0.0	Bias	0.00	X^b^	0.00	0.00	0.00	0.00	0.00	0.00	0.00	0.00	0.00
					–	0.00	0.00	0.00	0.00	0.00	0.00	0.00	0.00	0.00
			Variance (10^-3^)	1.670	X	1.686	1.691	1.679	1.683	1.676	1.676	1.678	1.679	1.677
					–	1.684	1.687	1.676	1.680	1.672	1.671	1.674	1.675	1.672
			MSE (10^-3^)	1.670	X	1.686	1.691	1.679	1.683	1.676	1.676	1.678	1.679	1.677
					–	1.684	1.687	1.676	1.680	1.672	1.671	1.674	1.675	1.672
			RRMSE (%)	16.3	X	16.4	16.4	16.4	16.4	16.4	16.4	16.4	16.4	16.4
					–	16.4	16.4	16.4	16.4	16.4	16.4	16.4	16.4	16.4
MAR 2	0.2	0.0	Bias	0.00	X^b^	0.00	0.00	0.00	0.00	0.00	0.00	0.00	0.00	0.00
					–	0.00	0.00	0.00	0.00	0.00	0.00	0.00	0.00	0.00
			Variance (10^-3^)	1.668	X	1.715	1.720	1.709	1.711	1.704	1.705	1.706	1.707	1.705
					–	1.684	1.689	1.678	1.682	1.673	1.668	1.674	1.676	1.670
			MSE (10^-3^)	1.669	X	1.715	1.720	1.709	1.711	1.705	1.706	1.707	1.708	1.706
					–	1.685	1.689	1.679	1.682	1.674	1.670	1.675	1.677	1.671
			RRMSE (%)	16.3	X	16.6	16.6	16.5	16.5	16.5	16.5	16.5	16.5	16.5
					–	16.4	16.4	16.4	16.4	16.4	16.3	16.4	16.4	16.4
MAR 3	0.5	0.0	Bias	0.00	X^b^	0.00	0.00	0.00	0.00	0.00	0.00	0.00	0.00	0.00
					–	0.00	0.00	0.00	0.00	0.00	0.00	0.00	0.00	0.00
			Variance (10^-3^)	1.761	X	1.992	2.002	1.981	1.985	1.973	1.977	1.978	1.977	1.976
					–	1.787	1.798	1.777	1.787	1.773	1.763	1.776	1.778	1.765
			MSE (10^-3^)	1.768	X	1.992	2.002	1.981	1.985	1.981	1.984	1.986	1.985	1.983
					–	1.793	1.804	1.784	1.793	1.781	1.771	1.784	1.786	1.773
			RRMSE (%)	16.8	X	17.9	17.9	17.8	17.8	17.8	17.8	17.8	17.8	17.8
					–	16.9	17.0	16.9	16.9	16.9	16.8	16.9	16.9	16.8

*Abbreviations*: *CCA* Complete case analysis, *IPPW* Inverse probability of participation weighting, *MSE* Mean square error, *RRMSE* Relative root mean square error

^a^Scenario MAR 1: response depending only on covariates; Scenarios MAR 2, 3: response depending on exposure and covariates

^b^Whether for the bias or the variance, the first line represents the result obtained when the exposure variable X is included in the response model, whereas the second line represents the result obtained without the exposure variable X in the response model

**Table 4:** Simulation study results: bias, variance, mean square error and related root mean square error in the $\hat{\beta}$ regression coefficient for CCA and the IPPW method (18 response models), for three MNAR response mechanism scenarios

Scenario^a^	***γ***_***x***_	***γ***_***y***_		CCA		IPPW method								
						*Response models*								
						(X), Z_1_, Z_2_, Z_3_, Z_4_	(X), Z_1_, Z_2_, Z_3_, Z_4_, Z_5_, Z_6_, Z_7_	(X), Z_1_, Z_3_	(X), Z_1_, Z_2_, Z_3_, Z_5_, Z_7_	(X), Z_1_	(X), Z_5_	(X), Z_1_, Z_5_	(X), Z_1_, Z_5_, Z_7_	(X), Z_5_, Z_7_
MNAR 1	0.0	0.2	Bias	0.00	X^b^	0.00	0.00	0.00	0.00	0.00	0.00	0.00	0.00	0.00
					–	0.00	0.00	0.00	0.00	0.00	0.00	0.00	0.00	0.00
			Variance (10^-3^)	1.676	X	1.701	1.707	1.694	1.698	1.688	1.685	1.689	1.690	1.686
					–	1.697	1.703	1.689	1.695	1.682	1.677	1.684	1.685	1.679
			MSE (10^-3^)	1.688	X	1.711	1.715	1.703	1.706	1.700	1.698	1.702	1.702	1.697
					–	1.709	1.714	1.702	1.706	1.695	1.690	1.697	1.698	1.691
			RRMSE (%)	16.4	X	16.5	16.6	16.5	16.5	16.5	16.5	16.5	16.5	16.5
					–	16.5	16.6	16.5	16.5	16.5	16.4	16.5	16.5	16.5
MNAR 2	0.2	0.2	Bias	−0.01	X^b^	−0.01	−0.01	−0.01	−0.01	−0.01	−0.01	−0.01	−0.01	−0.01
					–	− 0.01	− 0.01	− 0.01	− 0.01	− 0.01	− 0.01	− 0.01	− 0.01	− 0.01
			Variance (10^-3^)	1.668	X	1.746	1.751	1.737	1.740	1.728	1.727	1.730	1.730	1.728
					–	1.695	1.703	1.686	1.694	1.678	1.671	1.681	1.682	1.672
			MSE (10^-3^)	1.881	X	1.907	1.897	1.896	1.885	1.942	1.940	1.943	1.925	1.920
					–	1.904	1.907	1.900	1.900	1.892	1.884	1.895	1.896	1.885
			RRMSE (%)	17.3	X	17.5	17.4	17.4	17.4	17.6	17.6	17.6	17.5	17.5
					–	17.5	17.5	17.4	17.4	17.4	17.4	17.4	17.4	17.4
MNAR 3	0.5	0.2	Bias	−0.03	X^b^	−0.03	−0.02	−0.03	−0.02	−0.03	−0.03	−0.03	−0.03	− 0.03
					–	−0.03	− 0.03	− 0.03	− 0.03	−0.03	− 0.03	−0.03	− 0.03	−0.03
			Variance (10^-3^)	1.756	X	2.044	2.050	2.028	2.032	2.013	2.013	2.014	2.013	2.013
					–	1.793	1.806	1.780	1.794	1.772	1.760	1.777	1.778	1.761
			MSE (10^-3^)	2.572	X	2.708	2.650	2.685	2.630	2.885	2.882	2.885	2.811	2.806
					–	2.611	2.613	2.605	2.607	2.596	2.578	2.604	2.604	2.579
			RRMSE (%)	20.3	X	20.8	20.6	20.7	20.5	21.5	21.5	21.5	21.2	21.2
					–	20.4	20.4	20.4	20.4	20.4	20.3	20.4	20.4	20.3

*Abbreviations*: *CCA* Complete case analysis, *IPPW* Inverse probability of participation weighting, *MSE* Mean square error, *RRMSE* Relative root mean square error

^a^Scenarios MNAR 1: response depending on outcome and covariates; Scenario MNAR 2, 3: response depending on outcome, exposure, and covariates

^b^Whether for the bias or the variance, the first line represents the result obtained when the exposure variable X is included in the response model, whereas the second line represents the result obtained without the exposure variable X in the response model

**Table 5:** Simulation study results: bias, variance, mean square error and related root mean square error in the $\hat{\beta}$ regression coefficient for CCA and the IPPW method (18 response models), for three MNAR response mechanism scenarios

Scenario^a^	***γ***_***x***_	***γ***_***y***_		CCA		IPPW method								
						*Response models*								
						(X), Z_1_, Z_2_, Z_3_, Z_4_	(X), Z_1_, Z_2_, Z_3_, Z_4_, Z_5_, Z_6_, Z_7_	(X), Z_1_, Z_3_	(X), Z_1_, Z_2_, Z_3_, Z_5_, Z_7_	(X), Z_1_	(X), Z_5_	(X), Z_1_, Z_5_	(X), Z_1_, Z_5_, Z_7_	(X), Z_5_, Z_7_
MNAR 4	0.0	0.5	Bias	−0.02	X^b^	− 0.02	−0.01	− 0.01	− 0.01	− 0.02	− 0.02	−0.02	− 0.02	−0.02
					–	− 0.02	−0.02	− 0.02	− 0.02	−0.02	− 0.02	−0.02	− 0.02	−0.02
			Variance (10^-3^)	1.591	X	1.633	1.641	1.625	1.633	1.616	1.611	1.618	1.621	1.614
					–	1.620	1.630	1.613	1.623	1.603	1.594	1.607	1.610	1.597
			MSE (10^-3^)	1.857	X	1.859	1.849	1.849	1.840	1.885	1.878	1.887	1.866	1.852
					–	1.883	1.888	1.883	1.882	1.872	1.861	1.876	1.880	1.864
			RRMSE (%)	17.2	X	17.2	17.2	17.2	17.2	17.4	17.3	17.4	17.3	17.2
					–	17.4	17.4	17.4	17.4	17.3	17.3	17.3	17.3	17.3
MNAR 5	0.2	0.5	Bias	−0.04	X^b^	−0.04	−0.03	−0.04	−0.03	−0.04	−0.04	−0.04	− 0.04	− 0.04
					–	− 0.04	− 0.04	− 0.04	− 0.04	− 0.04	− 0.04	− 0.04	− 0.04	−0.04
			Variance (10^-3^)	1.621	X	1.737	1.743	1.727	1.731	1.712	1.708	1.711	1.712	1.709
					–	1.656	1.667	1.648	1.659	1.636	1.624	1.641	1.643	1.626
			MSE (10^-3^)	3.094	X	3.038	2.933	3.013	2.914	3.230	3.219	3.231	3.103	3.081
					–	3.142	3.137	3.144	3.138	3.125	3.104	3.136	3.140	3.108
			RRMSE (%)	22.3	X	22.0	21.7	22.0	21.6	22.7	22.7	22.7	22.3	22.2
					–	22.4	22.4	22.4	22.4	22.4	22.3	22.4	22.4	22.3
MNAR 6	0.5	0.5	Bias	−0.07	X^b^	−0.06	−0.06	−0.06	−0.06	−0.07	−0.07	−0.07	−0.07	−0.07
					–	−0.07	− 0.07	−0.07	− 0.07	−0.07	− 0.07	−0.07	− 0.07	−0.07
			Variance (10^-3^)	1.734	X	2.136	2.150	2.118	2.129	2.082	2.079	2.078	2.082	2.084
					–	1.786	1.810	1.770	1.798	1.755	1.742	1.764	1.767	1.744
			MSE (10^-3^)	6.211	X	6.284	5.938	6.229	5.896	6.903	6.887	6.901	6.503	6.473
					–	6.350	6.360	6.341	6.368	6.295	6.251	6.336	6.346	6.262
			RRMSE (%)	31.5	X	31.7	30.8	31.6	30.7	33.2	33.2	33.2	32.3	32.2
					–	31.9	31.9	31.9	31.9	31.7	31.6	31.8	31.9	31.7

*Abbreviations*: *CCA* Complete case analysis, *IPPW* Inverse probability of participation weighting, *MSE* Mean square error, *RRMSE* Relative root mean square error

^a^Scenarios MNAR 4: response depending on outcome and covariates; Scenario MNAR 5, 6: response depending on outcome, exposure, and covariates

^b^Whether for the bias or the variance, the first line represents the result obtained when the exposure variable X is included in the response model, whereas the second line represents the result obtained without the exposure variable X in the response model

**Table 6:** Simulation study results: coverage rate of the normality-based confidence interval for the $\hat{\beta}$ regression coefficient for CCA and the IPPW method (18 response models), for nine response mechanism scenarios

Scenario^a^	***γ***_***x***_	***γ***_***y***_	CCA		IPPW method								
					*Response models*								
					(X), Z_1_, Z_2_, Z_3_, Z_4_	(X), Z_1_, Z_2_, Z_3_, Z_4_, Z_5_, Z_6_, Z_7_	(X), Z_1_, Z_3_	(X), Z_1_, Z_2_, Z_3_, Z_5_, Z_7_	(X), Z_1_	(X), Z_5_	(X), Z_1_, Z_5_	(X), Z_1_, Z_5_, Z_7_	(X), Z_5_, Z_7_
MAR 1	0.0	0.0	94.9	X^b^	95.1	95.0	94.9	95.1	95.0	95.0	95.0	95.0	95.0
				–	95.1	95.0	94.9	95.0	94.9	95.0	95.0	94.9	95.0
MAR 2	0.2	0.0	95.1	X^b^	95.1	95.1	95.1	95.0	95.2	95.2	95.1	95.1	95.2
				–	95.0	95.0	95.0	95.0	95.0	95.2	95.0	95.0	95.1
MAR 3	0.5	0.0	94.9	X^b^	95.0	94.9	95.0	95.0	94.8	94.9	94.8	94.9	94.9
				–	94.8	94.9	94.8	94.9	94.9	94.9	94.9	94.9	95.0
MNAR 1	0.0	0.2	95.0	X^b^	95.2	95.2	95.1	95.1	95.0	95.0	95.0	95.0	95.1
				–	95.2	95.2	95.1	95.1	94.9	95.0	95.0	95.0	95.0
MNAR 2	0.2	0.2	93.8	X^b^	94.1	94.3	94.2	94.3	93.8	93.8	93.7	94.0	93.9
				–	93.8	94.0	93.9	93.8	93.8	93.8	93.8	93.9	93.8
MNAR 3	0.5	0.2	89.9	X^b^	91.5	91.9	91.5	92.0	90.2	90.3	90.1	90.6	90.8
				–	89.9	90.0	89.8	90.0	89.7	90.0	89.6	89.6	89.9
MNAR 4	0.0	0.5	93.2	X^b^	93.6	93.6	93.7	93.7	93.3	93.3	93.3	93.3	93.4
				–	93.4	93.3	93.3	93.3	93.2	93.1	93.2	93.2	93.2
MNAR 5	0.2	0.5	84.3	X^b^	86.2	87.0	86.2	87.0	84.4	84.5	84.4	85.4	85.5
				–	84.3	84.6	84.1	84.3	84.3	84.3	84.2	84.2	84.3
MNAR 6	0.5	0.5	63.9	X^b^	71.2	73.4	71.2	73.5	66.5	66.8	66.5	69.0	69.4
				–	64.2	64.7	63.9	64.3	63.6	63.6	63.7	63.5	63.4

### Bias in the $\hat{\boldsymbol{\beta}}$ regression coefficient

We observed no bias with either CCA or the IPPW method for the three MAR scenarios and MNAR scenario 1 (***γ***_***x***_ = **0.0**, ***γ***_***y***_ = **0.2**). A bias occurred with both methods for the five other MNAR scenarios, with a greater amplitude for MNAR scenarios 5 (***γ***_***x***_ = **0.2**, ***γ***_***y***_ = **0.5**) and 6 (***γ***_***x***_ = **0.5**, ***γ***_***y***_ = **0.5**). The bias was globally equivalent between CCA and the IPPW method for these five scenarios. Within the IPPW method, all response models tested showed the same bias pattern across all MAR and MNAR scenarios. For MNAR scenarios 1 to 3, the absolute bias increases as ***γ***_***x***_ increases. Similarly, the absolute bias increases as ***γ***_***x***_ increases for MNAR scenarios 4 to 6.

### Variance of the $\hat{\boldsymbol{\beta}}$ regression coefficient

**Fig. 2:**
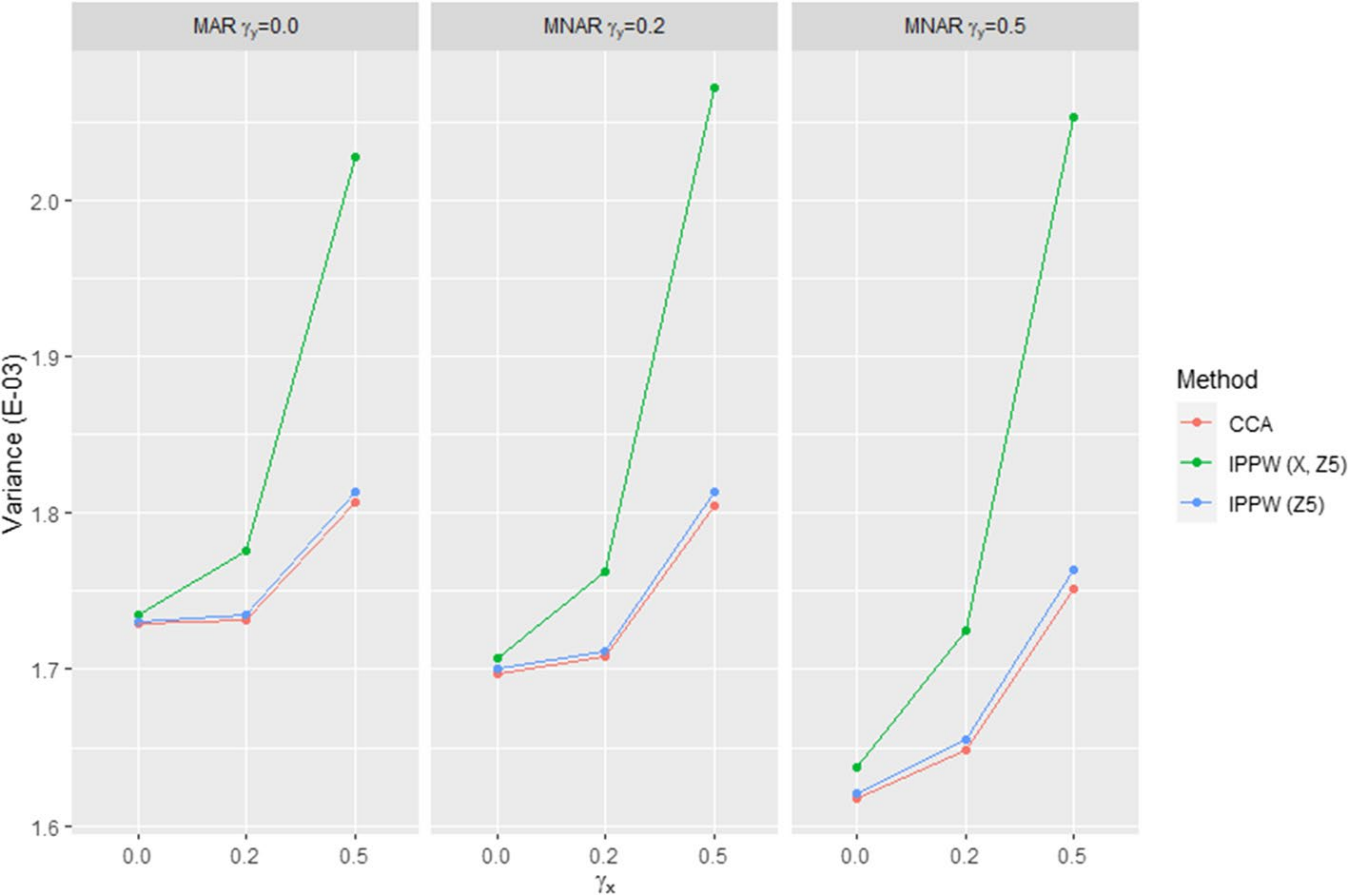
Monte-Carlo variance obtained with CCA and the IPPW method (response models 1 and 10, see Table 2) for the nine response mechanism scenarios. The variance increased as the correlation between the exposure variable and the response variable increased for both methods. The variance was consistently higher with the IPPW method than with CCA in all scenarios. With the IPPW method, variance inflation was particularly observed when the exposure variable X was put into the response model

**Fig. 3:**
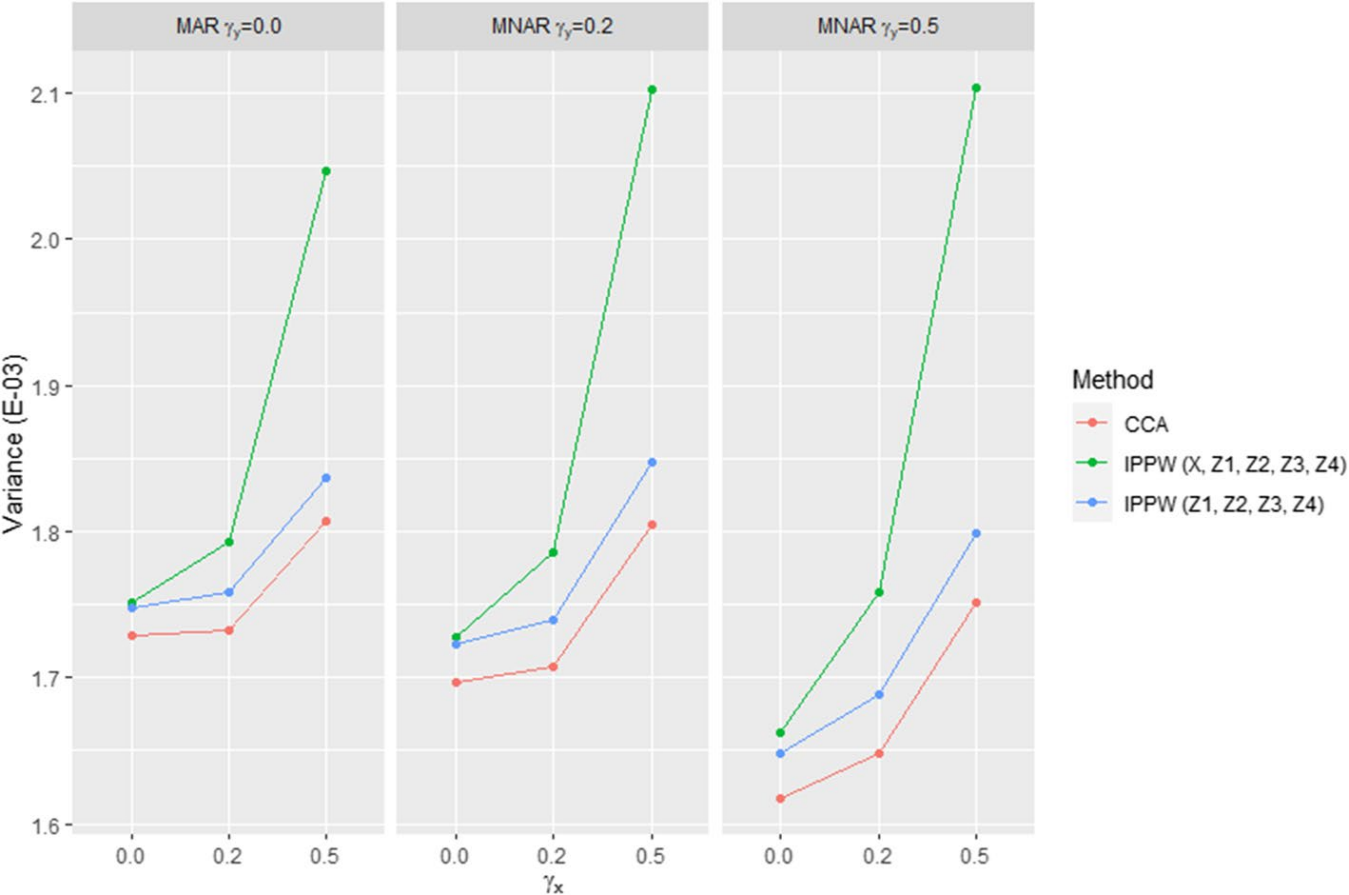
Monte-Carlo variance obtained with CCA and the IPPW method (response models 6 and 15, see Table 2) for the nine response mechanism scenarios. The variance increased as the correlation between the exposure variable and the response variable increased for both methods. The variance was consistently higher with the IPPW method than with CCA in all scenarios. With the IPPW method, variance inflation was particularly observed when the exposure variable X was put in the response model. On the other hand, removal of variable X (covariate Z_5_ only) resulted in the variance obtained with the IPPW method being very close to that obtained by CCA

The IPPW method was less efficient than CCA for all scenarios. We observed an increase in variance with increasing correlation between the exposure variable X and the response (illustrated by Figs. 2 and 3). The loss of efficiency of the IPPW method was thus particularly pronounced in MAR scenario 3 and MNAR scenarios 3 and 6 (all three characterized by ***γ***_***x***_ = **0.5**).

The response models that enabled a reduction in the variance were those in which the exposure variable X was removed (see Figs. 2 and 3). This was particularly observed in scenarios in which the exposure variable X was associated with the response, but it was also observed in MAR scenario 1 and MNAR scenarios 1 and 4 (all three characterized by ***γ***_***x***_ ***=*** **0.0**). Among the response models without the exposure variable X, the response model that further reduced the variance was that which included only the variable Z_5_ (confounding variable not associated with the response). Nevertheless, response models including the variables Z_5_, Z_7_ (a confounding variable and a prognostic variable, neither associated with the response), and only Z_1_ (confounding variable associated with the response) also showed good performance in terms of precision. Overall, the gain in precision obtained with all these response models did not enable us to reach the level of precision obtained using CCA.

### Mean square error of the $\hat{\boldsymbol{\beta}}$ regression coefficient

For the MAR scenarios, all the tested estimators are unbiased and there is therefore no difference between the variance and the mean square error (see Table 3). For MNAR scenarios 1 to 3, the mean square error increases with ***γ***_***x***_, i.e. when the correlation between the exposure variable and the response increases. This also holds true for MNAR scenarios 4 to 6.

### Coverage rates

The coverage rates are well respected for all the MAR scenarios and for MNAR scenario 1. For MNAR scenarios 1 to 3, the coverage decreases as the correlation between the exposure variable and the response increases. This also holds true for MNAR scenarios 4 to 6. The coverage rates are poorly respected for MNAR scenarios 5 and 6.

## Illustrative example

As an example, we analyzed the association between pre-pregnancy maternal BMI with the child’s BMI at age 7 in TIMOUN, a prospective mother-child cohort study conducted in the Guadeloupe archipelago (French West Indies) [16].

### Study population and data collection

Between November 2004 and December 2007, 1068 pregnant women were enrolled in TIMOUN by obstetricians during their second- or third-trimester prenatal visit at public hospitals or at a local dispensary. At inclusion, women were interviewed by trained midwives to assess their medical history, socioeconomic conditions, and lifestyle. At birth, information concerning maternal diseases during pregnancy, health status of the newborn, and details of the delivery was also collected [17]. In total, 1033 single live births were registered. Several follow-ups were organized within a selected subsample of the children at 3, 7, and 18 months of age [18, 19]. When the children were 7 years of age, all the mothers initially included were invited to participate in a new follow-up which consisted of an interview of the mothers and a medical examination of the children. Among the 1033 mother-child couples initially included, 592 participated in this second wave, representing 57% of the initial sample. Weight was not measured for two children examined at age 7, resulting in a final population of 590 for the association studied (see detailed flow-chart in Additional file 1: e-Appendix 2).

### Outcome and exposure

The exposure of interest was the pre-pregnancy maternal BMI (kg/m^2^). It was calculated from the mothers’ self-reported weight and height before pregnancy at inclusion in the cohort. The outcome of interest was the child’s BMI at 7 years. It was calculated from measurements performed during a medical examination at 7 years.

### Covariates

The covariates considered in the analysis were maternal age at birth (continuous), maternal educational level (< 5 years, 5–12 years, > 12 years), maternal place of birth (French West Indies, other Caribbean island, Europe), non-gestational maternal diabetes (yes, no), enrollment site (university hospital, local hospital, antenatal care dispensary), maternal alcohol consumption during pregnancy (yes, no), maternal smoking during pregnancy (yes, no), sex of the child (boy, girl).

The proportion of missing data within these covariates did not exceed 3%, except for maternal alcohol consumption during pregnancy (5.6%). For the variables with missing values, a single imputation by the modal value was previously performed.

**Fig. 4:**
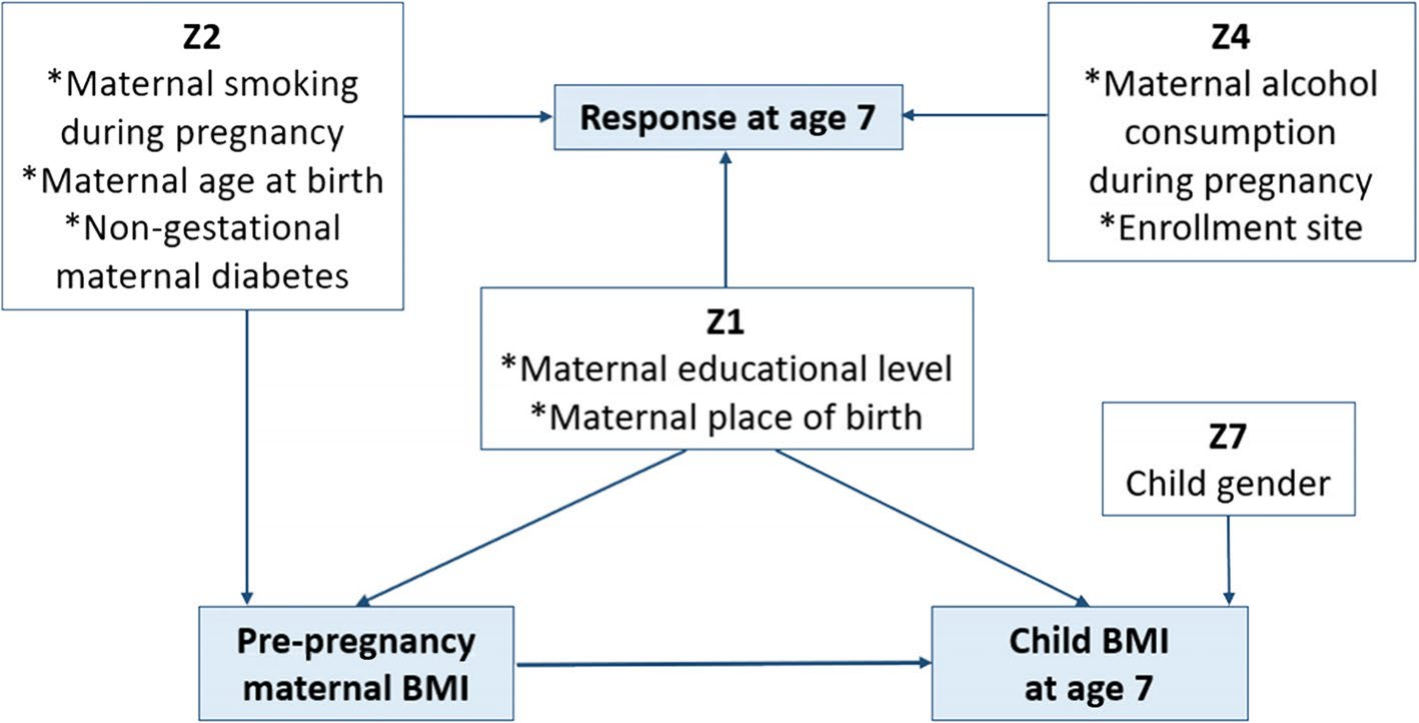
Directed acyclic graph (DAG) of the known or assumed associations between variables of the illustrative example. For the sake of simplicity and clarity, the arrows representing the associations between the covariates are not drawn. Not all types of variables considered in the simulation study were suitable for this illustrated example. The covariates ‘maternal educational level’ and ‘maternal place of birth’ were considered to be confounding factors in the relationship between pre-pregnancy maternal BMI and child BMI at age 7. The association models were therefore adjusted for these covariates

The directed acyclic graph (DAG) on which we based our analyses is presented in Fig. 4. All arrows were placed according to a priori knowledge. In our study, the DAG approach did not identify all the types of covariates Z presented in the simulation study: no variables of type Z_3_, Z_5_, or Z_6_ were present in our example. In this didactic example, we assume a situation equivalent to the MAR 1 scenario in the simulation study (i.e. the response at 7 years depends neither on the exposure nor the outcome, but only on the covariates).

A linear regression model was fitted with an a priori adjustment for maternal education and maternal place of birth (confounding variables). Both CCA and the IPPW method were applied, the latter using several response models.

The analyses were performed using R 3.3.2 (R Foundation for Statistical Computing, Vienna, Austria). The standard errors were computed taking into account the weight estimation phase, according to the method described by Metten et al. [20].

The R code to implement the CCA and IPPW analyses and a training dataset are available in Additional file 1: e-Appendix 3.

## Results

The β coefficients related to the exposure of interest were very similar between CCA and the IPPW method (Table 7). Within the IPPW results, the most effective response model strategy was the one including only Z_1_ variables (maternal educational level and maternal place of birth).

**Table 7:** Adjusted association between pre-pregnancy maternal BMI and child BMI at age 7 (CCA and IPPW method)

	β	SE
CCA (*N* = 590)^a^	0.142	0.0197
IPPW method (*N* = 590)^a^		
*Response models*		
Z_1_, Z_2_, Z_4_	0.137	0.0229
Z_1_, Z_2_, Z_4_, Z_7_	0.138	0.0231
Z_1_, Z_2_, Z_7_	0.140	0.0234
Z_1_	0.140	0.0224

Z_1_: maternal educational level and maternal place of birth

Z_2_: maternal tobacco smoking during pregnancy, maternal age at birth, and non-gestational maternal diabetes

Z_4_: enrollment site and maternal alcohol consumption during pregnancy

Z_7_: sex of the child

*Abbreviations*: *CCA* Complete case analysis, *IPPW* Inverse probability of participation weighting, *β* Beta coefficient (regression estimate), *SE* Standard error

^a^Adjustment for maternal educational level and maternal place of birth

## Discussion

Attrition is a major methodological issue in cohort studies. It challenges the validity of association analyses because its occurrence is generally not completely at random. Several authors have proposed the IPPW method to correct for potential selection biases [3, 4]. However, little evaluation of the method has been performed and there is little guidance for researchers who wish to apply it, in particular for the specification of the response model.

Our simulation study showed no superiority of the IPPW method over CCA in terms of bias, and it even led to a loss of efficiency. Both were similarly unbiased in the MAR scenarios and similarly biased in most MNAR scenarios. For the MNAR scenarios, the absolute bias increased as the correlation between the exposure and the response increased. As a result, the mean square error is high for these scenarios when ***γ***_***x***_ = **0.5**. In addition, because the bias is negative, the confidence intervals are shifted to the left and the nominal error rates are poorly respected.

These results are consistent with those observed in a study comparing several methods of handling attrition in a simulated cohort of 300 subjects [21]. In this study, the authors concluded that CCA produces results as valid as those obtained with the other compared methods, which included the IPPW method. It is worth noting that the IPPW method consists in reweighting the study population with complete data, meaning that both CCA and IPPW methods are based on the same sub-population. Therefore, a difference in efficiency cannot be attributed to a varying sample size. One explanation for the loss of efficiency observed with the IPPW method lies in the fact that adding covariates in the response model tends to increase the variability of estimated weights.

We chose to solely compare the IPPW method to CCA. However, there are also other approaches, including imputation methods, which consist of replacing missing values with plausible ones. Multiple imputation (MI) is an advanced imputation method that has steadily improved and gained popularity in recent years [22, 23]. It consists of imputing the dataset several times by using adapted models that include the collected variables. However, imputation methods are mainly used for missing covariates in situations of partial non-response. Seaman and White emphasized that it may be potentially dangerous to use MI in situations of total non-response [4]. The risk of mis-specifying the imputation model would be high because it requires the imputation of all missing variables of a given survey wave, without auxiliary information at the time of the survey wave. The results of Lewin et al. also showed that MI was no better than CCA in situations of attrition that lead to a missing outcome [24]. This is also consistent with the findings of Kristman et al. [21].

Our study aimed also to assess the impact of the choice of the variables included in the response model on the bias of the exposure regression coefficient and its variance. The various response models tested did not change the bias patterns, which is consistent with what has been observed in the literature on the propensity score method. Indeed, Brookhart et al. showed that the issue of the choice of variables resided essentially in the variance, not in the bias [12]. The strategy of not including variables associated with the exposure in the propensity score, but rather confounding and prognostic variables, improved the precision of the estimates without increasing the bias.

In our study, we show that it is preferable not to include the exposure variable in the response model. Otherwise variance inflation would be observed, which is not in line with the proposal of Hernan et al. [3]. Paradoxically, this phenomenon was particularly pronounced in scenarios in which the exposure variable was associated with the response. This can possibly be explained by over-fitting because the exposure variable is present in the association model.

Within response models without the exposure variable, the minimalist strategy, consisting of including only the confounding variable unrelated to the response, resulted in the lowest estimated variance. Close response models (in order of best precision: inclusion of both confounding and prognostic variables unrelated to the response; inclusion of the confounding variable related to the response) also performed well in terms of precision. Thus, parsimonious strategies using the same variables as the association model (except the exposure variable) were the most effective. This was also observed in our illustrated example. The strategy to optimize the response model when using the IPW method to limit a selection bias (IPPW) is thus the same as that recommended in situations in which the IPW method is used to limit a confounding bias (propensity scores).

The strategy for constructing the response model requires clear identification of the role played by the variables. This can be based on a structural approach using DAGs, as we did in our example in Section 5 [3]. DAGs are causal analysis tools originally designed to assist in the selection of variables in an association model [25]. They make it possible to control for a confusion bias and avoid over-adjustment in the association model. Within the framework of the propensity score method, Austin and Stuart recommended using them to identify sets of variables to be included in the propensity score [26]. Similarly, it can be useful to guide the variable selection in the response model. Indeed, it allows researchers to better visualize the relationships between all the variables involved in the association of interest (exposure, outcome, response, covariates) and thus enables optimization of the specification of the response and association models.

DAGs are based on a priori knowledge and thus do not protect against misidentification of the role played by the variables. In surveys, rather than weighting individuals by their individual probability of response, the sample is often partitioned into response homogeneity groups (RHGs), i.e. groups that are homogeneous in terms of response probability. The parameters of interest are estimated in each group and then pooled across the groups to obtain an overall parameter. This strategy, while improving the precision of the estimates, protects against possible misspecification of the response model. The RHG method is quite similar to what is referred to as stratification in the context of the propensity score used to reduce a confounding bias. In the context of attrition, Seaman and White proposed a stratified IPPW method, but the stratification was not based on response probability but rather response patterns to survey waves [4]. Once the strata were defined, a response model was fitted independently in each stratum. We are not aware of any use of stratification on the probability of response in association studies based on cohort data, but this may be a new application of an existing method in other contexts (surveys, and propensity scores).

### Strengths

The first strength of our study is that we tested through simulations nine response mechanism scenarios, corresponding to three degrees of correlation between the response variable and our interest variables (exposure, outcome). The parameters chosen were consistent with those observed in the TIMOUN cohort to represent a realistic setting. In addition, we evaluated the impact of several response models on the estimated exposure effect. This has not been previously performed in the literature when the IPW method has been used to reduce a selection bias.

### Limitations

This study also had limitations. First, our simulation framework did not consider binary outcomes, although this is a common situation in epidemiology. However, recent literature indicates that CCA is potentially much less prone to give biased estimates of the exposure coefficient in a logistic regression [27], making the linear regression framework more challenging for evaluating the IPPW method. Second, in our simulations the level of attrition was kept constant, at a quite high but realistic level (40%) for cohort studies. The influence of the attrition rate is however well known, with the expected conclusion that bias and variance increase with the percentage of non-respondents [21]. Moreover, the attrition level we chose was realistic for cohort studies and thus quite high (40%). Third, we did not vary the degree of correlation between the covariates Z and the response or our variables of interest (exposure, outcome). However, Lewin et al. showed that a strong correlation between the outcome and a variable of type Z_3_ (i.e. associated with the outcome and response) could increase the magnitude of the bias [24]. Finally, our study only considered attrition leading to missing outcomes and fully observed exposure and covariates. However, these variables may also be affected by partial non-response in everyday practice. Seaman proposed a mixed approach to address this problem, combining MI and the IPPW method [28]. Although it has already been used by several epidemiological researchers [29–31], such an approach should be further explored, especially for the evaluation of its superiority over a method combining IPPW and single imputation.

Finally, we did not address the consequence of using *estimated* weights (inverse response probability) in the association models. The usual statistical packages and procedures estimate the standard error of the effect of exposure as if weights are a priori known, ignoring the extra-variability due to their estimation. Consequently, the standard error is biased and may mislead the conclusions about the significance of the effect. We proposed an exact estimation of the variance (linearized variance) that should be used when IPW is implemented (as we did in our illustrative example). The details of the calculations of this variance are available in Metten et al. [20].

## Conclusion

Our study suggests that using IPPW to handle attrition in cohort studies does not reduce bias and may result in a loss of efficiency. These results therefore raise questions about the contribution of the IPW method to correcting possible selection bias that occurs in situations of attrition that lead to a missing outcome in association analyses. If the method is to be used, we encourage use of only the confounding variables of the association of interest in the response model.

## Supplementary Information

**Additional file 1: e-Appendix 1.** SAS code to implement the simulation study. **e-Appendix 2.** Detailed flow-chart of the TIMOUN cohort. **e-Appendix 3.** R Script to implement an IPPW analysis - Illustrative example (demo dataset “dt.csv”).

## Data Availability

The SAS code corresponding to the simulation study, the R code to implement an IPPW analysis, and a training dataset are provided in the Additional file 1 of the article. The dataset from the TIMOUN cohort used as an illustrative example in this article cannot be made openly available due to ethical concerns. The TIMOUN team can provide the data on request, after appropriate approvals. Requests should be submitted to Dr. Luc Multigner.
