# Supplementary material for: Inverse probability weighting to handle attrition in cohort studies: some guidance and a call for caution
Source: BMC Med Res Methodol. 2022 Feb 16;22:45. doi: 10.1186/s12874-022-01533-9 (PMC8848672; doi:10.1186/s12874-022-01533-9)
Supplement: Supplementary file 1 — Additional file 1: e-Appendix 1. SAS code to implement the simulation study. e-Appendix 2. Detailed flow-chart of the TIMOUN cohort. e-Appendix 3. R Script to implement an IPPW analysis - Illustrative example (demo dataset “dt.csv”). [file 12874_2022_1533_MOESM1_ESM.docx]

**e-Appendix : Inverse probability weighting to handle attrition in cohort studies: some guidance and a call for caution**

Metten, MA, Costet N, Multigner L, Chauvet G

**Table of Contents**

e-Appendix 1: SAS code to implement the simulation study

e-Appendix 2: Detailed flow-chart of the TIMOUN cohort

e-Appendix 3: R Script to implement an IPPW analysis - Illustrative example (demo dataset “dt.csv”)

**e-Appendix 1: SAS code to implement the simulation study**

This script corresponds to the simulation study presented in the paper, leading to the simulation results presented in Tables 3a-3c and in Table 4.

/************************************************************/

/* 29/11/2021 : simulation program for the revised paper */

/************************************************************/

/* Variables : */

/* -> Z_1,...,Z_7 ~ N(0,1) independent */

/* -> Exposition variable X=f(Z_1,Z_2,Z_5,Z_6) */

/* Correlations of 0.2 between X and Z_1, Z_2, Z_5, Z_6 */ /* -> Outcome variable Y=f(X,Z_1,Z_3,Z_5,Z_7) */ /* Correlation of 0.3 between Y and X */ /* Correlations of 0.2 between Y and Z_1, Z_3, Z_5, Z_7 */ /* -> Response variable R=f(X,Y,Z_1,Z_2,Z_3,Z_4) */ /* Coefficient of X equal to 0, 0.2 or 0.5 */ /* Coefficient of Y equal to 0, 0.2 or 0.5 */ /* Coefficients of Z_1, Z_2, Z_3, Z_4 equal to 0.1 */ /* Mean response probability of approximately 60 % */ /* Sample size : n=1,000 */ /************************************************************/

/* Response model */ /* R=f() */ /* R=f((X),Z1,Z2,Z3,Z4) */ /* R=f((X),Z1,Z2,Z3,Z4,Z5,Z6,Z7) */ /* R=f((X),Z1, ,Z3) */ /* R=f((X),Z1,Z2,Z3, ,Z5, ,Z7) */ /* R=f((X),Z1) */ /* R=f((X), ,Z5) */ /* R=f((X),Z1, ,Z5) */ /* R=f((X),Z1, ,Z5, ,Z7) */ /* R=f((X), ,Z5, ,Z7) */ /************************************************************/

/* Association model */ /* Y=f(X Z1 Z5 ) */ /************************************************************/

libname base "...";

/************************************************************/

/* Some SAS IML tools */ /* -> wgtlog : weighted logistic regression */ /* -> wgtreg : weighted linear regression */

/************************************************************/

**%macro** ***outils***;

/*******************************************************/

/* Weighted logistic regression (Newton method) */ /* Input : */ /* -> outcome variable r(n,1) */ /* -> covariates x(n,q) */ /* -> weighting variable w(n,1) */ /* Output : */ /* -> Estimated parameters alpha(q,1) */ /*******************************************************/

start wgtlog(r=,x=,w=);

q=ncol(x);alpha=J(q,**1**,**0**);f=**1**;

maxiter=**20**;converge=**0.000001**;

do iter=**1** to maxiter while(max(abs(f))>converge);

p=exp(x*alpha)/(**1**+exp(x*alpha));

f=((w#(r-p))#x)[+,];f=t(f);

J=-t(x)*(w#p#(**1**-p)#x);

delta = -solve(J,f);

alpha = alpha+delta;

end;

return (alpha);

finish wgtlog;

/**************************************/

/* Weighted linear regression */

/* Input : */

/* -> outcome variable y(n,1) */

/* -> covariates z(n,p) */

/* -> individual variance v(n,1) */

/* -> weighting variable w(n,1) */

/* Output : */

/* -> Estimated parameters beta(p,1) */

/**************************************/

start wgtreg(y=,z=,v=,w=);

beta=inv(t(z)*((w/v)#z))

*(t(z)*((w/v)#y));

return (beta);

finish wgtreg;

**%mend** outils;

/************************************************************/

/* Monte Carlo simulations */

/************************************************************/

**%macro** varsim(iter=**10000**,nech=**1000**,base=work,lib=base,

sort1=sort_00_00,sort2=sort_02_00,sort3=sort_05_00,sort4=sort_00_02,sort5=sort_02_02,sort6=sort_05_02,sort7=sort_00_05,sort8=sort_02_05,sort9=sort_05_05);

data ech;do i=**1** to &nech;output;end;run;

%do i=**1** %to &iter;

%put Itération &i;

/********************/

/* Data generation */

/********************/

data ech;set ech;

/* Covariates */

z1=rannor(**0**);z2=rannor(**0**);z3=rannor(**0**);

z4=rannor(**0**);z5=rannor(**0**);z6=rannor(**0**);

z7=rannor(**0**);

/* Exposition variable */

x=**1**+**0.218***z1+**0.218***z2+**0.218***z5+**0.218***z6+rannor(**0**);

/* Outcome */

y=**1**+**0.250***x+**0.230***(z3+z7)+**0.170***(z1+z5)+rannor(**0**);

/* Response probabilities */

u=ranuni(**0**);un=**1**;

p1=**1**/(**1**+exp(-(+**0.40**+**0.0***x+**0.0***y+**0.1***(z1+z3)+**0.1***z2+**0.1***z4)));

p2=**1**/(**1**+exp(-(+**0.23**+**0.2***x+**0.0***y+**0.1***(z1+z3)+**0.1***z2+**0.1***z4)));

p3=**1**/(**1**+exp(-(-**0.05**+**0.5***x+**0.0***y+**0.1***(z1+z3)+**0.1***z2+**0.1***z4)));

p4=**1**/(**1**+exp(-(+**0.15**+**0.0***x+**0.2***y+**0.1***(z1+z3)+**0.1***z2+**0.1***z4)));

p5=**1**/(**1**+exp(-(-**0.02**+**0.2***x+**0.2***y+**0.1***(z1+z3)+**0.1***z2+**0.1***z4)));

p6=**1**/(**1**+exp(-(-**0.28**+**0.5***x+**0.2***y+**0.1***(z1+z3)+**0.1***z2+**0.1***z4)));

p7=**1**/(**1**+exp(-(-**0.18**+**0.0***x+**0.5***y+**0.1***(z1+z3)+**0.1***z2+**0.1***z4)));

p8=**1**/(**1**+exp(-(-**0.35**+**0.2***x+**0.5***y+**0.1***(z1+z3)+**0.1***z2+**0.1***z4)));

p9=**1**/(**1**+exp(-(-**0.63**+**0.5***x+**0.5***y+**0.1***(z1+z3)+**0.1***z2+**0.1***z4)));

r1=(u<=p1);r2=(u<=p2);r3=(u<=p3);r4=(u<=p4);r5=(u<=p5);

r6=(u<=p6);r7=(u<=p7);r8=(u<=p8);r9=(u<=p9);

run;

proc iml;

use ech;

read all var{un} into un;

read all var{un} into covr1;

read all var{un x z1 z2 z3 z4} into covr2;

read all var{un z1 z2 z3 z4} into covr3;

read all var{un x z1 z2 z3 z4 z5 z6 z7} into covr4;

read all var{un z1 z2 z3 z4 z5 z6 z7} into covr5;

read all var{un x z1 z3 } into covr6;

read all var{un z1 z3 } into covr7;

read all var{un x z1 z2 z3 z5 z7} into covr8;

read all var{un z1 z2 z3 z5 z7} into covr9;

read all var{un x z1 } into covr10;

read all var{un z1 } into covr11;

read all var{un x z5 } into covr12;

read all var{un z5 } into covr13;

read all var{un x z1 z5 } into covr14;

read all var{un z1 z5 } into covr15;

read all var{un x z1 z5 z7} into covr16;

read all var{un z1 z5 z7} into covr17;

read all var{un x z5 z7} into covr18;

read all var{un z5 z7} into covr19;

read all var{y} into y;

read all var{un x z1 z5 } into covy1;

close ech;

%***outils***;

/* Response mechanism */

%do j=**1** %to **9**;

use ech;read all var{r&j} into r;close ech;

/* Association model */

%do k=**1** %to **1**;

/* Response model */

%do l=**1** %to **19**;

a=wgtlog(r,covr&l,un);

phat=exp(covr&l*a)/(**1**+exp(covr&l*a));

w=r/phat;

b=wgtreg(y,covy&k,un,w);

b1=b1||b[**2**];

%end;

%end;

%if %sysfunc(exist(&lib.**.**&&sort&j)) %then %do;

edit &lib.**.**&&sort&j;append from b1;close &lib.**.**&&sort&j;

%end;

%else %do;

create &lib.**.**&&sort&j from b1;append from b1;close &lib.**.**&&sort&j;

%end;

free b1;

%end;

quit;

%end;

**%mend** varsim;

option nonotes;

%***varsim***(iter=**10000**,nech=**1000**,base=work,

lib=base,

sort1=sort_00_00,sort2=sort_02_00,sort3=sort_05_00,

sort4=sort_00_02,sort5=sort_02_02,sort6=sort_05_02,

sort7=sort_00_05,sort8=sort_02_05,sort9=sort_05_05);

option notes;

/************************************************************/

/* Computation of the bias, variance, MSE and RRMSE */ /* Three tables : */ /* -> MAR */ /* -> moderate MNAR */ /* -> strong MNAR */ /************************************************************/

**%macro** mef(tab=sort_00_00,indic=indic_00_00,

indic2=sort_00_00);

proc means data=base.&tab noprint;var col1-col19;

output out=&indic

%do i=**1** %to **18**;

mean(col&i)=m_col&i var(col&i)=v_col&i

%end;

mean(col19)=m_col19 var(col19)=v_col19;

run;

data &indic;set &indic;

%do i=**1** %to **19**;

b_col&i=m_col&i-**0.250**;

mse_col&i=b_col&i****2**+v_col&i;

rmse_col&i=sqrt(mse_col&i)/**0.250**;

%end;

run;

data ligne1;set &indic(keep=b_col1 b_col2 b_col4 b_col6 b_col8 b_col10 b_col12 b_col14 b_col16 b_col18);

rename b_col1=col1

b_col2=col2

b_col4=col3

b_col6=col4

b_col8=col5

b_col10=col6

b_col12=col7

b_col14=col8

b_col16=col9

b_col18=col10;

run;

data ligne1b;set &indic(keep=b_col1 b_col3 b_col5 b_col7 b_col9 b_col11 b_col13 b_col15 b_col17 b_col19);

rename b_col1=col1

b_col3=col2

b_col5=col3

b_col7=col4

b_col9=col5

b_col11=col6

b_col13=col7

b_col15=col8

b_col17=col9

b_col19=col10;

run;

data ligne2;set &indic(keep=v_col1 v_col2 v_col4 v_col6 v_col8 v_col10 v_col12 v_col14 v_col16 v_col18);

rename v_col1=col1

v_col2=col2

v_col4=col3

v_col6=col4

v_col8=col5

v_col10=col6

v_col12=col7

v_col14=col8

v_col16=col9

v_col18=col10;

run;

data ligne2b;set &indic(keep=v_col1 v_col3 v_col5 v_col7 v_col9 v_col11 v_col13 v_col15 v_col17 v_col19);

rename v_col1=col1

v_col3=col2

v_col5=col3

v_col7=col4

v_col9=col5

v_col11=col6

v_col13=col7

v_col15=col8

v_col17=col9

v_col19=col10;

run;

data ligne3;set &indic(keep=mse_col1 mse_col2 mse_col4 mse_col6 mse_col8 mse_col10 mse_col12 mse_col14 mse_col16 mse_col18);

rename mse_col1=col1

mse_col2=col2

mse_col4=col3

mse_col6=col4

mse_col8=col5

mse_col10=col6

mse_col12=col7

mse_col14=col8

mse_col16=col9

mse_col18=col10;

run;

data ligne3b;set &indic(keep=mse_col1 mse_col3 mse_col5 mse_col7 mse_col9 mse_col11 mse_col13 mse_col15 mse_col17 mse_col19);

rename mse_col1=col1

mse_col3=col2

mse_col5=col3

mse_col7=col4

mse_col9=col5

mse_col11=col6

mse_col13=col7

mse_col15=col8

mse_col17=col9

mse_col19=col10;

run;

data ligne4;set &indic(keep=rmse_col1 rmse_col2 rmse_col4 rmse_col6 rmse_col8 rmse_col10 rmse_col12 rmse_col14 rmse_col16 rmse_col18);

rename rmse_col1=col1

rmse_col2=col2

rmse_col4=col3

rmse_col6=col4

rmse_col8=col5

rmse_col10=col6

rmse_col12=col7

rmse_col14=col8

rmse_col16=col9

rmse_col18=col10;

run;

data ligne4b;set &indic(keep=rmse_col1 rmse_col3 rmse_col5 rmse_col7 rmse_col9 rmse_col11 rmse_col13 rmse_col15 rmse_col17 rmse_col19);

rename rmse_col1=col1

rmse_col3=col2

rmse_col5=col3

rmse_col7=col4

rmse_col9=col5

rmse_col11=col6

rmse_col13=col7

rmse_col15=col8

rmse_col17=col9

rmse_col19=col10;

run;

data &indic2;set ligne1 ligne1b ligne2 ligne2b ligne3 ligne3b ligne4 ligne4b;run;

**%mend** mef;

%***mef***(tab=sort_00_00,indic=indic_00_00,indic2=indic2_00_00);

%***mef***(tab=sort_02_00,indic=indic_02_00,indic2=indic2_02_00);

%***mef***(tab=sort_05_00,indic=indic_05_00,indic2=indic2_05_00);

%***mef***(tab=sort_00_02,indic=indic_00_02,indic2=indic2_00_02);

%***mef***(tab=sort_02_02,indic=indic_02_02,indic2=indic2_02_02);

%***mef***(tab=sort_05_02,indic=indic_05_02,indic2=indic2_05_02);

%***mef***(tab=sort_00_05,indic=indic_00_05,indic2=indic2_00_05);

%***mef***(tab=sort_02_05,indic=indic_02_05,indic2=indic2_02_05);

%***mef***(tab=sort_05_05,indic=indic_05_05,indic2=indic2_05_05);

**data** tab1;set indic2_00_00 indic2_02_00 indic2_05_00;**run**;

**data** tab2;set indic2_00_02 indic2_02_02 indic2_05_02;**run**;

**data** tab3;set indic2_00_05 indic2_02_05 indic2_05_05;**run**;

/************************************************************/

/* Coverage rate (95 % nominal level confidence interval) */ /************************************************************/

**%macro** coverage(tab=sort_00_00,cov=cov_00_00);

proc means data=base.&tab noprint;var col1-col19;

output out=vmc var=;

run;

data vmc;set vmc;

%do i=**1** %to **19**; call symput("vmc&i",col&i); %end;

run;

data tab;set base.&tab;

%do i=**1** %to **19**;

cov&i=(col&i-**1.96***sqrt(&&vmc&i)<=**0.250**)*(col&i+**1.96***sqrt(&&vmc&i)>=**0.250**); %end;

run;

proc means data=tab noprint;var cov1-cov19;output out=&cov(drop=_type_ _freq_) mean=;run;

**%mend** coverage;

%***coverage***(tab=sort_00_00,cov=cov_00_00);

%***coverage***(tab=sort_02_00,cov=cov_02_00);

%***coverage***(tab=sort_05_00,cov=cov_05_00);

%***coverage***(tab=sort_00_02,cov=cov_00_02);

%***coverage***(tab=sort_02_02,cov=cov_02_02);

%***coverage***(tab=sort_05_02,cov=cov_05_02);

%***coverage***(tab=sort_00_05,cov=cov_00_05);

%***coverage***(tab=sort_02_05,cov=cov_02_05);

%***coverage***(tab=sort_05_05,cov=cov_05_05);

**data** export_cov;

set cov_00_00 cov_02_00 cov_05_00

cov_00_02 cov_02_02 cov_05_02

cov_00_05 cov_02_05 cov_05_05;

**run**;

**e-Appendix 2: Detailed flow-chart of the TIMOUN cohort**

**
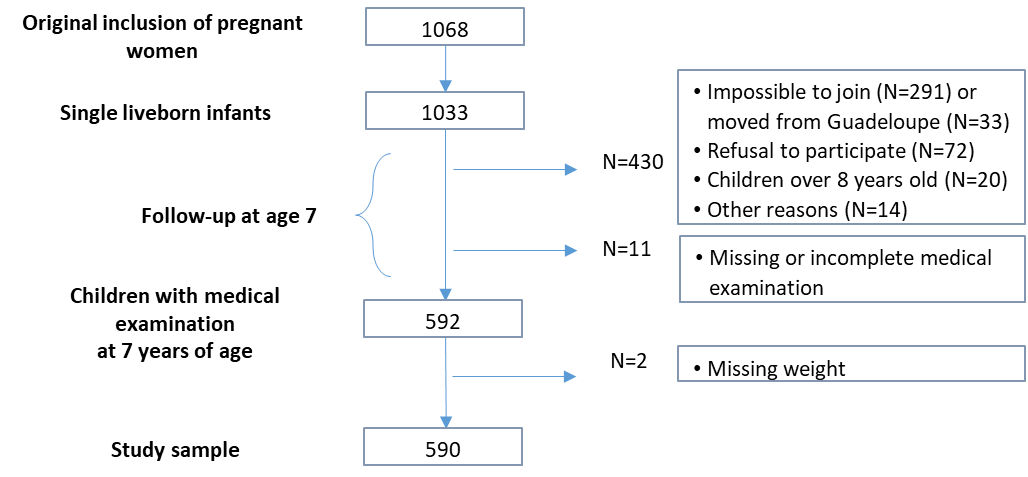
**

**e-Appendix 3: R Script to implement an IPPW analysis: Training example (demo dataset “dt.csv”)**

This script is based on the illustrative example presented in Section 5 and applicable to the demo dataset “dt.csv”. The association of interest is between the maternal pre-pregnancy body mass index (BMI) and the child’s BMI at 7 years of age, within a mother-child cohort.

The database contains 200 observations, including 79 non-participants at age 7 (attrition rate = 39.5%). For these 79 children, the outcome of interest (BMI at 7 years) is unknown.

The exposure variable and the covariates were fully observed at inclusion in the cohort (baseline).

**Variable names**

***Response variable (measured at 7 years)***

Missing_7: missing at age 7 (binary)

***Exposure variable (measured at inclusion in the cohort)***

Maternal_BMI: maternal body mass index before pregnancy

***Outcome variables (measured at 7 years)***

BMI_7: child’s BMI at age 7 (continuous)

Overweight_7: overweight child at age 7 (binary)

***Covariates (measured at inclusion in the cohort)***

Maternal_education: maternal educational level (3 levels)

Maternal_origin: maternal place of birth (3 levels)

Sex: sex of the child (binary)

Smoking_pregnancy: maternal smoking during pregnancy (binary)

Maternal_age: maternal age at birth (continuous)

Maternal_diabetes : non-gestational maternal diabetes (binary)

Enrollment_site: center (3 levels)

Alcohol pregnancy: maternal alcohol consumption during pregnancy (binary)

##################

**## Data import**

##################

dt<-read.csv(file="Path/dt.csv", sep=",")

##################

**## Complete-case analysis (CCA)**

##################

# Association model: Linear regression

mod_cca <- lm(BMI_7 ~ Maternal_BMI + Maternal_education + Maternal_origin, data=dt)

summary(mod_cca)

# Association model: Logistic regression

mod_cca <- glm(tot_merge$Overweight_7 ~ Maternal_BMI + Maternal_education + Maternal_origin, data=dt, family="binomial")

summary(mod_cca)

############

**## Implementation of IPPW method**

############

#P_hat: response probabilities (estimated from the response model)

P_hat <- predict(glm(Missing_7 ~ Maternal_education + Maternal_origin + Smoking_pregnancy

+ Maternal_age + Maternal_diabetes + Enrollment_site + Alcohol_pregnancy,

data=dt , family="binomial"), type="response")

# Association model: weighted linear regression

mod_ipw_lm <- lm(BMI_7 ~ Maternal_BMI + Maternal_education + Enrollment_site, data=dt, weights=1/P_hat)

summary(mod_ipw_lm)

# Association model: weighted logistic regression

mod_ipw_glm <- glm(Overweight_7 ~ Maternal_BMI + Maternal_education + Maternal_origin,data=dt, weights=1/P_hat, family=quasibinomial)

summary(mod_ipw_glm)
